# Computed tomography measurements of different dimensions of maxillary and frontal sinuses

**DOI:** 10.1186/1471-2342-11-8

**Published:** 2011-04-05

**Authors:** Pernilla Sahlstrand-Johnson, Magnus Jannert, Anita Strömbeck, Kasim Abul-Kasim

**Affiliations:** 1 Department of Oto-Rhino-Laryngology, Faculty of Medicine, Lund University, Skåne University Hospital, Malmö, Sweden; 2 Division of Neuroradiology, Diagnostic Centre for Imaging and Functional Medicine, Faculty of Medicine, Lund University, Skåne University Hospital, Malmö, Sweden

## Abstract

**Background:**

We have previously proposed the use of Doppler ultrasound to non-invasively stage sinus infection, as we showed that acoustic streaming could be generated in nonpurulent sinus secretions and helped to distinguish it from mucopurulent sinus secretions. In order to continue this development of a clinically applicable Doppler equipment, we need to determine different dimensions of the paranasal sinuses, especially the thickness of the anterior wall of the maxillary sinus (at the canine fossa). To the best of our knowledge, this is the first report on the thickness of the canine fossa. This study aimed to (a) estimate different dimensions of the maxillary and frontal sinuses measured on computed tomography (CT) of the head, (b) define cut-off values for the normal upper and lower limits of the different measured structures, (c) determine differences in age, side and gender, (d) compare manually and automatically estimated maxillary sinuses volumes, and (e) present incidental findings in the paranasal sinuses among the study patients.

**Methods:**

Dimensions of 120 maxillary and frontal sinuses from head CTs were measured independently by two radiologists.

**Results:**

The mean value of the maxillary sinus volume was 15.7 ± 5.3 cm^3 ^and significantly larger in males than in females (P = 0.004). There was no statistically significant correlation between the volume of maxillary sinuses with age or side. The mean value of the bone thickness at the canine fossa was 1.1 ± 0.4 mm. The automatically estimated volume of the maxillary sinuses was 14-17% higher than the calculated volume. There was high interobserver agreement with regard to the different measurements performed in this study. Different types of incidental findings of the paranasal sinuses were found in 35% of the patients.

**Conclusion:**

We presented different dimensions of the maxillary and frontal sinuses on CTs. We believe that our data are necessary for further development of a clinically applicable Doppler equipment for staging rhinosinusitis.

## Background

The paranasal sinuses are complex anatomical structures with a significant inter-individual variation. The use of computed tomography (CT) instead of plain radiography in the work-up of paranasal sinus pathology was recommended in the beginning of the 1990's [1]. Since then CT has become mandatory in the preoperative work-up of sinus surgery. In addition, CT has become an essential aid in navigation during the functional endoscopic sinus surgery (FESS).

The different anatomical dimensions of the paranasal sinuses can also be obtained from CT images. Kawarai's report on volume quantification of the paranasal sinuses on three-dimensional CT scans [2] was followed by different studies as this technique has been continuously developed and improved [3-5]. Although there are published studies on the anatomy of the paranasal sinuses, there are still dimensions of the maxillary sinus and the surrounding structures that need to be investigated.

We have previously demonstrated the potential for a new application of the Doppler ultrasound technique that makes it possible to determine the properties of paranasal sinus fluids safely and non-invasively [6,7]. Our findings have supported the hypothesis that nonpurulent sinus secretions (which have a low viscosity) can be distinguished from mucopurulent sinus secretions (which have a high viscosity) with Doppler ultrasound, since acoustic streaming can be generated and detected in serous sinus fluid but not in mucopurulent sinus secretions [6]. Consequently, this method has the potential to improve the diagnosis of rhinosinusitis and potentially imply a decrease of the prescription of antibiotics. In order to continue the development of this new Doppler application, we needed to estimate the anatomical dimensions of the maxillary and frontal sinuses. The delivery of acoustic intensity into the sinus cavity is highly dependant on the thickness of the anterior bony wall of the sinuses. In addition, we have established that the choice of radius of the ultrasound beam is dependant on the radius of the sinus cavity [7].

Upon performing Doppler ultrasound, the probe is usually placed on the patient's cheek at the level of the nostril at the canine fossa, which is a rounded depression below the infraorbital foramen where levator anguli oris muscle originate. Bovine bone samples with a thickness of 1.08 ± 0.7 mm were used in our experimental studies to mimic the anterior wall of the maxillary sinus [7]. However, the choice of bone thickness in those experiments was based on surgical experience, as we were not able to find any published data on the thickness of the bone in this area in human series. Subsequently, clinically relevant dimensions of the paranasal sinuses and the adjacent soft tissue are still lacking. As the maxillary sinuses are situated just below the orbit, separated only by a thin bony wall, the knowledge of the anatomical dimensions is also of importance when considering safety aspects of the usage of Doppler ultrasound in this area. To the best of our knowledge, this is the first report in literature on the thickness of the bony and the soft tissue structures at the canine fossa. Moreover, there is no comparison in the literature between manually and automatically estimated sinus volumes. As manual estimation of sinus volume is easier to perform and less time consuming, finding a quotient to manually measure the maxillary sinus volume would be advantageous.

The aims of this study were to estimate different dimensions of the maxillary and frontal sinuses measured on head CTs, define a cut-off values for the normal upper and lower limits of the different measured structures, and to find out if age, side or gender of the individuals had any correlation with the different measured structures. Furthermore, we compared manually and automatically estimated sinus volume of the maxillary sinuses, and we aimed to define a quotient that helps a manual estimation of the maxillary sinus volume. Finally, we present the incidental findings in the paranasal sinuses among the study patients.

## Methods

**Figure 1:**
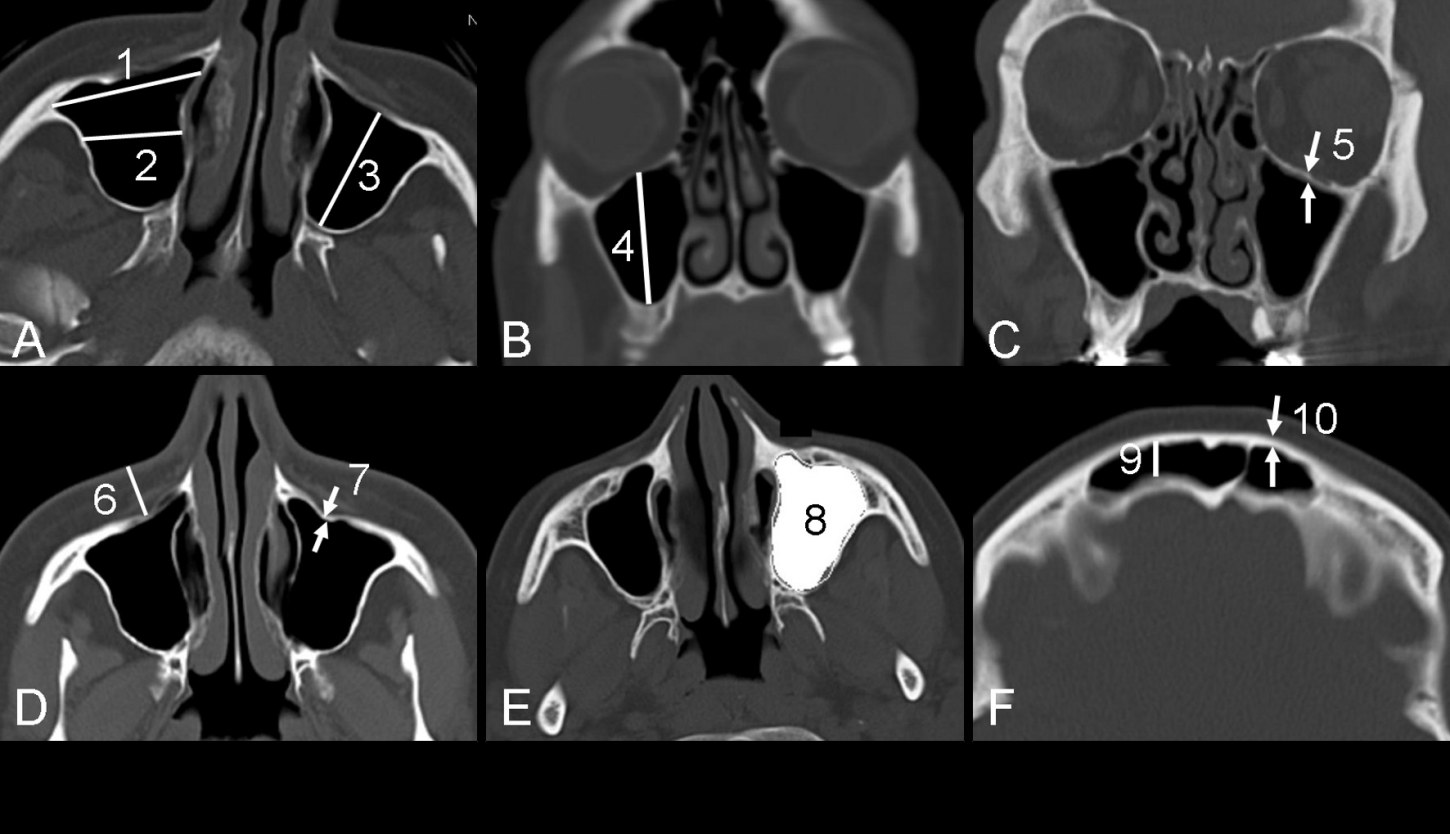
**(A, D-F) axial CT images and (B-C) coronal CT image showing the way of the measurement of different dimensions**: (1) maximal width of the maxillary sinus, (2) the width at the middle of the maxillary sinus, (3) maximal depth (anteroposterior diameter) of the maxillary sinus, (4) maximal height of the maxillary sinus, (5) thickness of the orbital floor, (6) the thickness of soft tissue between the anterior wall of maxillary sinus at the canine fossa and the skin surface, (7) the thickness of anterior wall of the maxillary sinus (canine fossa), and (8) the automatically measured volume. (9) The depth of the frontal sinus, and (10) the thickness of the anterior wall of the frontal sinus were measured at the level of the orbital roof, 1 cm lateral to the midline. All measurements were made on images with skeletal settings.

Head CTs of 60 consecutive patients (32 females and 28 males) with mean age of 40 ± 14 years (median 41 and range 18-65 years) were included in this retrospective analysis. The Local Ethics Committee of Lund approved the study protocol. The patient's age was equally distributed with 20 patients in each age group: 18-32, 33-49, and 50-65 years. All patients were examined on a multislice CT scanner (SOMATOM Sensation 16, Siemens AG, Forchheim, Germany). The indications for Head CTs were: trauma (n = 16), headache (n = 16), neurological deficit and stroke (n = 13), epilepsy (n = 5), vertigo (n = 5), others (N = 5: visual disturbance, facial pain, tinnitus, anosmia, nausea). Patients with midfacial injuries were excluded. No patients with tumor, mucocele or evidence of previous sinus surgery were found among the patients included in the analyses of this study. Images were obtained with slice collimation of 0.75 mm. Axial and coronal images with slice thickness of 4.5 and 3 mm, respectively using skeletal algorithm and skeletal window (window center 700 and window width 2600) were used for analysis in the Picture Archive and Communication System (PACS, SECTRA). The following measurements were performed independently by two neuroradiologists:

(1) Maxillary sinuses: (a) maximal craniocaudal diameter, (b) maximal depth (anteroposterior diameter), (c) maximal width, (d) the width at the middle of the maxillary sinus on the axial slices, and (e) the thickness of the bony anterior wall (canine fossa). The latter was measured 1.5 cm below margo infraorbitalis (the proposed positions of the canine fossa). Measurement (a) was performed on coronal images whereas the remaining measurements were performed on axial images.

(2) The thickness of soft tissue between the anterior wall of maxillary sinus at the canine fossa and the skin surface was measured on the axial images at the same level as (1e).

(3) Frontal sinuses: (a) maximal depth (anteroposterior diameter), and (b) the thickness of anterior wall were measured on the axial images at the level of the orbital roof.

(4) Thickness of the orbital floor was measured on the coronal images. Figure 1 shows the way of performing the above mentioned measurements.

The given value for every measurement was the mean value of the measurements obtained by the two readers. Furthermore, the volume of the maxillary sinuses was estimated at the Leonardo work station (Siemens AG, Medical Solutions, Erlangen, Germany) using the volume application (figure 1). The estimation of the attenuation of the gas in the maxillary sinus enabled an automatic estimation of the volume of each maxillary sinus for every individual patient included in the study. Maxillary sinuses with mucous membrane swelling, cysts and/or fluid was subjected to 2-step estimation of their total volume by estimation of the volume of the gas containing portion followed by estimation of the volume of the consolidated portion of the sinus. The latter was estimated after measuring the attenuation of the structures filling the sinus. The range of the attenuation of the gas in the maxillary sinuses was set to -200 to -1200 HU. The mean value for the attenuation of the gas of the maxillary sinuses was -892 ± 226 HU (range -701 to -914 HU).

As all three dimensions of the maxillary sinus were measured, the volume of each maxillary sinus was also calculated using the following equation: (Width × anteroposterior × craniocaudal diameter × 0.5). The width used for this calculation was the mean value for the maximal width and the width at the middle of the maxillary sinus on the axial slices (measurements marked 1 and 2 in figure 1a).

### Statistical analysis

Statistical analysis was performed with SPSS 17 (originally; Statistical Package for the Social Sciences). Data is presented as proportions (%) or as mean with 95% confidence interval (95% CI) or with standard deviations (SD). Reliability analysis of the interobserver agreement with regard to the different performed measurements was done by: (1) calculating a two-way mixed model of intraclass correlation coefficient (ICC), and (2) performing a paired sample t-test to calculate the random errors for the differences. The random error was the SD of the interobserver differences of each measurement. The interpretations of the ICC were done according to the one proposed by Landis and Koch [8,9] A kappa of 1 indicates total agreement whereas a kappa of zero means poor agreement and indicates that any observed agreement is attributed to chance. A kappa of 0.81-1.00 indicates almost perfect agreement, 0.61-0.80 indicates substantial agreement, 0.41-0.60 indicates moderate agreement, 0.21-0.40 indicates fair agreement, 0-0.20 indicates slight agreement, and a kappa of <0 indicates poor agreement.

Mann-Whitney U test was performed to test the association between the different measurements of the maxillary and frontal sinuses on one hand and the different categorical variables. Spearman's correlation was used to test the correlation of the same measurements with continuous variables. Differences with a *P *value ≤ 0.05 were considered statistically significant.

## Results

The results of the reliability analysis of CT as a method for the measurement of different dimensions of maxillary and frontal sinuses and other nearly related structures are shown in Table 1. The intraobserver agreement was almost perfect in the estimation of the craniocaudal diameter of maxillary sinuses, the depth of the frontal sinuses, and the anterior wall of frontal sinuses, and substantial in the estimation of the anteroposterior diameter of maxillary sinuses and the thickness of the canine fossa. The interobserver agreement in the estimation of the anterior wall of maxillary sinuses and the orbital floor was moderate with interobserver random error for differences in the measurement of these structures varying between 0.3 and 0.5 millimeter (Table 1).

**Table 1:** Reliability analysis showing interobserver agreement in the measurements of different anatomical structures expressed as intraclass correlation coefficient (ICC).

	ICC (95% CI)		Random error, mm	
	**Right**	**Left**	**Right**	**Left**

**Maxillary sinus**:				
Craniocaudal diameter	0.88 (0.80-0.92)	0.87 (0.80-0.92)	2.4	2.5
A-P diameter	0.79 (0.68-0.87)	0.79 (0.67-0.87)	2.7	2.8
Anterior wall thickness	0.58 (0.38-0.73)	0.59 (0.39-0.73)	0.4	0.4

**Frontal sinus**:				
A-P diameter	0.80 (0.68-0.88)	0.86 (0.77-0.91)	2.1	1.8
Anterior wall thickness	0.84 (0.74-0.90)	0.87 (0.79-0.92)	0.5	0.4

**Canine fossa**:				
AP-diameter	0.73 (0.58-0.83)	0.75 (0.62-0.85)	2.6	2.7

**Orbital floor**:				
Thickness	0.50 (0.29-0.67)	0.57 (0.37-0.72)	0.3	0.3

The table shows the random error of interobserver differences, expressed in millimeter.

95% CI indicates 95% confidence interval.

A-P diameter indicates anteroposterior diameter (depth).

The mean values of the different measured dimensions were not correlated to the patient's age (correlation coefficient 0.126, P = 0.172). The median value for the estimated volumes of the maxillary sinuses in patients of different age groups were 14.4 cm^3 ^for patients aged 18-33 years, 16.6 cm^3 ^for patients aged 34-49 years and 15.2 cm^3 ^for patients aged 50-65 years (P = 0.299). The mean values for the volume and the craniocaudal diameter of maxillary sinuses as well as the anteroposterior diameter of the frontal sinus of male patients were significantly greater than the corresponding values for female patients (Table 2). The mean value, SD and median value of the volume of the maxillary sinuses of both sides were 15.7, 5.3, and 15.2 cm^3^, respectively. The volume of the maxillary sinuses of both sides was significantly greater in male patients than in female patients (median 18 vs. 14.1 cm^3^, P = 0.004). There was no statistically significant difference between the estimated volume of the right and the left sided maxillary sinuses (median 15.3 vs. 15.5 cm^3^, P = 0.727). The mean value, SD and median value of the bony anterior wall of the maxillary sinus at the canine fossa of both sides were 1.1, 0.4, and 1 mm, respectively. There was no significant difference in anterior wall thickness of the frontal sinuses between the sexes (Table 2). Additionally, there was neither any gender difference in soft tissue thickness between anterior wall of maxillary sinus and the skin surface, nor in thickness of the orbital floor.

**Table 2:** shows female:male distribution of the mean value, SD, median value, range and normal cut-off values of the measurements of different anatomical structures.

	study cohort	Female			Male			P-value
	**Mean ± SD**	**Mean ± SD** **(median)**	**Range**	**Normal** **values**	**Mean ± SD** **(median)**	**Range**	**Normal** **values**	

**Maxillary sinus**:								
Volume (right)	15.4 ± 5	14 ± 3 (14)	5-19	8-20	18 ± 6 (18)	9-32	6-30	**0.002**
Volume (left)	16 ± 6	15 ± 4 (15)	7-21	7-23	18 ± 7 (18)	7-34	4-32	**0.016**
Craniocaudal diameter (right)	31.3 ± 5	30 ± 3 (31)	20-35	24-36	34 ± 5 (33)	27-43	24-44	**0.004**
Craniocaudal diameter (left)	31.3 ± 5	30 ± 3 (30)	24-34	24-36	33 ± 5 (34)	21-43	23-43	**0.020**
A-P diameter (right)	35 ± 4	35 ± 3 (35)	27-41	29-41	36 ± 3 (36)	31-46	30-42	0.056
A-P diameter (left)	35.6 ± 4	34 ± 4 (34)	27-40	26-42	35 ± 4 (36)	26-43	27-43	0.058
Width (right)	23.4 ± 4	23 ± 3 (22)	12-28	17-29	25 ± 4 (25)	18-34	17-33	**0.018**
Width (left)	23.7 ± 4	23 ± 3 (24)	16-30	17-29	25 ± 5 (25)	14-33	15-35	0.125
Anterior wall thickness at canine fossa (right)	1.1 ± 0.4	1 ± 0.4 (1)	0.6-2.3	0.2-1.8	1.1 ± 0.3 (1.2)	0.6-2.1	0.5-1.7	0.266
Anterior wall thickness at canine fossa (left)	1.1 ± 0.4	1.1 ± 0.4 (1)	0.6-2.5	0.3-1.9	1 ± 0.3 (1)	0.5-1.8	0.4-1.6	0.504

**Frontal sinus**:								
A-P diameter (right)	9.6 ± 3	9 ± 4 (9)	4-20	1-17	10 ± 3 (10)	6-16	4-16	**0.034**
A-P diameter (left)	10.2 ± 3.3	9 ± 3 (9)	5-20	3-15	11 ± 3 (11)	6-18	5-17	**0.046**
Anterior wall thickness (right)	2.1 ± 0.8	2 ± 0.6 (1.9)	1.2-3.5	0.8-3.2	2.1 ± 1 (1.9)	0.9-5.2	0.1-4.1	0.824
Anterior wall thickness (left)	2.1 ± 0.8	2.1 ± 0.7 (1.9)	0.8-4.1	0.7-3.5	1.9 ± 0.7 (1.7)	0.9-3.7	0.5-3.5	0.450

**Soft tissue thickness between anterior wall of maxillary sinus and the skin surface**								
AP-diameter (right)	11.6 ± 3	11 ± 3 (11)	5-15	5-17	12 ± 4 (11)	6-20	4-20	0.300
AP-diameter (left)	12.1 ± 4	11 ± 4 (11)	5-18	3-19	13 ± 4 (12)	5-21	5-21	0.227

**Orbital floor**								
Thickness (right)	0.9 ± 0.2	0.8 ± 0.2 (0.8)	0.5-1.4	0.4-1.2	0.9 ± 0.3 (0.8)	0.4-1.4	0.3-1.5	0.440
Thickness (left)	0.9 ± 0.2	0.9 ± 0.3 (0.9)	0.5-2	0.3-1.5	0.9 ± 0.3 (0.8)	0.5-1.4	0.3-1.5	0.853

SD indicates standard deviation.

The volume is given in cm^3 ^whereas other values are given in millimeter.

P-values of statistically significant female:male differences are written in bold style.

Normal values: The lower limit equals mean -2SD whereas the upper limit equals mean +2SD.

A-P diameter indicates anteroposterior diameter (depth).

**Figure 2:**
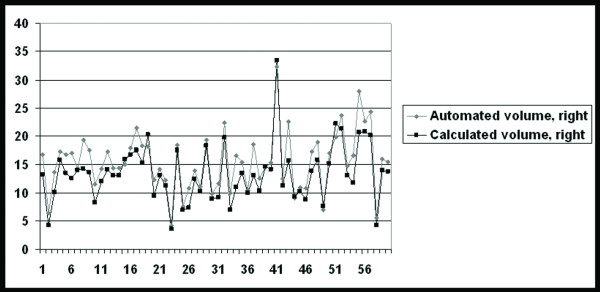
**Diagram showing the automated and the calculated volume of the right maxillary sinus**. Note that the automated volume exceeded the calculated volume in 52 out of 60 cases. The volume is presented in cm^3 ^on the y-axis and the patient's number is presented on the x-axis.

The degree of agreement between the automated measurement of the volume of maxillary sinuses and the volume calculated according to the equation width × anteroposterior × craniocaudal diameter × 0.5, was almost perfect (ICC 0.90-0.93 and random error of 1.9-2.4 cm^3^). In 52 patients the automatically estimated volume was in average 14-17% greater than the calculated volume in the right sided maxillary sinuses (Figure 2).

In five out of 60 patients (8.3%) the frontal sinuses were not pneumatisized. Seventeen out of 120 maxillary sinuses (14%) subjected for automatic volume measurements, exhibited a volume < 10 cm^3^. Maxillary sinuses with volume < 10 cm^3 ^was found in 11 out of 60 patients (18%) (bilateral in six patients and unilateral in the remaining five). Only one maxillary sinus exhibited a volume < 5 cm^3^.

Twenty-one patients (35%) showed different types of incidental findings of paranasal sinuses. These are summarized in Table 3.

**Table 3:** summarizes the incidental findings of the paranasal sinuses among the study patients.

	Right (No.)	Left (No.)	Bilateral (No.)	Total (No.)
Maxillary sinuses:				
-Non-specific MMS	5	2	1	8
-Fluid	2			2
-Retention cysts		1	2	3

Frontal sinuses: MMS			1	1

Sphenoidal sinuses: MMS		1	1	2

Ethmoidal sinuses: MMS		1	4	5

Total				21

MMS indicates mucous membrane swelling

## Discussion

This study has shown that CT is a robust method in the estimation of different dimensions of the maxillary sinuses, frontal sinuses and the adjacent structures as the interobserver agreement ranges from substantial to almost perfect dependent on the measurement in question (Table 1). Despite the moderate interobserver agreement with regard to the measurements of the canine fossa and the orbital floor (ICC ranging between 0.50 and 0.60), the random error was only 0.3-0.4 mm. This depends partly on the fact that these structures are very thin and partly on the limitation of measurements of tiny structures in PACS. The results in the present study are of importance when setting the adjustments of a clinical applicable Doppler ultrasound equipment for the diagnose of rhinosinusitis.

Previous studies have shown that dimensions of maxillary sinuses from measurements on human skulls were similar to those obtained by CT scans [10] and the consistency of measurements of the paranasal sinuses using CT images have been evaluated in the last decade [2,5,10]. Some authors have measured the volume by directly injecting different materials into the paranasal sinuses [11,12]. However, this procedure cannot be used in living subjects. Furthermore, using such methods in the estimation of the sinus volume usually result in underestimation of the volume in the presence of mucosal thickening and other sinus pathologies [5,11,12]. Our analysis was performed on head CT in patients subjected to trauma, and patients with headache, neurological deficit and stroke, epilepsy, and vertigo. Thus, our material represents individuals with no history of sinus pathology and can in practice be considered as "normal population".

The results of the maxillary sinuses measurements were consistent with previous reports [2,5]. The mean values of the maxillary sinus volume have been reported to range from 11.1 ± 4.5 cm^3 ^to 23.0 ± 6.7 cm^3 ^in previous studies [5].

We found that there was a significant difference of the maxillary sinus volume between males and females, mainly due to the fact that male exhibit higher and wider maxillary sinuses than females. Similarly, the anteroposterior diameter of the frontal sinus was larger in men. Some authors have reported difference of the volume of the maxillary sinuses between males and females [2,4,13] whereas others have showed no such difference [5]. Ariji et al have described the correlation between the craniocaudal diameter of the maxillary sinus and body height, body weight and age [10]. As men are generally larger than women, this could explain our observed difference in gender for maxillary sinus volume.

In our work we only included adults (age 18-65 years) and we found neither significant age difference nor significant difference between the left and right maxillary sinus volume. Previous reports suggested that the maxillary sinus volume increase with both age [14] and loss of teeth [15]. On the other hand Ariji found no significant difference between dentate and edentulous patients [10].

To our knowledge this is the first report on the thickness of the canine fossa. The bone thickness was 1.1 mm (mean value for study cohort), which correlates well with our surgical experience or when inspecting dried skulls. The thickness of the soft tissue in front of the bone of the canine fossa varied from 5 to 20 mm. These results are of special importance in our future work with the evaluation of Doppler ultrasound as a diagnostic tool for staging rhinosinusitis, as bone attenuates ultrasound waves considerably, and soft tissue does not. The volume and anteroposterior diameter of the sinuses are also relevant for the development of this new Doppler application, as we previously showed that the radius of the ultrasound transducer should correspond to half the radius of the sinus cavity [7]. This novel application of the Doppler ultrasound technique makes it possible to determine the properties of paranasal sinus fluids safely and non-invasively. It has previously been proved that the Doppler ultrasound technique can be used to identify mucopurulent rhinosinusitis [6]. This method could improve the diagnosis of rhinosinusitis, reduce the suffering of patients with rhinosinusitis and potentially decrease the prescription of antibiotics. This in turn would lead to a decrease in antibiotic resistance and a significant cost reduction for the health care services as a whole.

In our study we measured the anteroposterior diameter and the anterior wall thickness of the frontal sinuses at the level of the orbital roof (Figure 1F). We chose this reference point since one upon performing an ultrasound examination of the frontal sinuses usually hold the ultrasound probe against this area and it is consequently the dimensions of this area that affect the ultrasound wave of the prospective Doppler equipment. The thickness of the bone of the anterior wall of the frontal sinuses is approximately twice as thick as the anterior bony wall of the maxillary sinus in our material, which implies that the attenuation of the ultrasound waves would be much higher when examining the frontal sinuses. Subsequently, it would be hard to induce acoustic streaming in secretions in the frontal sinuses. The anteroposterior diameter of the frontal sinus at this reference point may not be the deepest of the frontal sinuses and consequently our data are difficult to compare to results of other authors.

This study showed a good concordance between the manual and automatically calculated volume of the maxillary sinus with ICC ranging between 0.90 and 0.93. The results from the automatically computed data were 14-17% higher than the manually calculated volumes, which enable a rough estimation of the maxillary sinus volume by measuring the sinus diameter in three planes. Although such estimation is not suitable for research purposes, we believe that this tool might be beneficial in clinical practice for approximate estimation of the maxillary sinus volume, where volume measurement applications are not available.

In our study, there were incidental findings of the paranasal sinuses in 35% of the patients which correlates well to previous reports, where mucosal changes in the paranasal sinuses have been detected in 17-42.5% of CT scans for non-rhinological disease [16-18]. Non-specific mucosal swelling was the commonest finding (27% of the patients) in our material, whereas the incidence of maxillary mucosal cysts was less frequent than previously reported (12.4 to 22%) [19,20].

The measurements of this study were done by two radiologists. One of the drawbacks of this study was that some selection bias might have occurred by the subjective selection of the slice by each reader. However, the reader's choice of the slice should have been almost identical to give such a good agreement in most of the measurements that is shown in Table 1. Other drawbacks are the retrospective nature of the study and inclusion of patients rather than healthy individuals. However, the radiation doses of head CT amounts to 2-2.5 mSv, which make the exposure of healthy individuals to such high radiation doses ethically unacceptable.

## Conclusions

This study showed that CT is a reliable method for the measurement of different dimensions of the maxillary and frontal sinus. We presented data on the thickness of canine fossa, which is not previously studied or reported to our knowledge. We believe that these data are necessary for further development of a clinically applicable Doppler equipment for staging a sinus infection. Furthermore, we showed a good correlation between the manually and the automatically estimated maxillary sinuses volumes. Finally, we have described incidental findings in the paranasal sinuses, which is of importance when interpreting CT scans in patients with possible rhinosinusitis.

## Competing interests

The authors declare that they have no competing interests.

## Authors' contributions

PSJ has contributed to conception and design of the study, analysis and interpretation of data, drafting the manuscript and has given her final approval of the version to be published. MJT has contributed to the revision of the manuscript critically for important intellectual content, and has given his final approval of the version to be published. ASK has contributed to analysis and interpretation of data, drafting the manuscript and has given her final approval of the version to be published. KAK has contributed to conception and design of the study, acquisition of data, analysis and interpretation of data, drafting the manuscript and has given his final approval of the version to be published. All four authors have read and approved the final manuscript.

## Pre-publication history

The pre-publication history for this paper can be accessed here:

http://www.biomedcentral.com/1471-2342/11/8/prepub
